# Mindfulness training induces structural connectome changes in insula networks

**DOI:** 10.1038/s41598-018-26268-w

**Published:** 2018-05-21

**Authors:** Paul B. Sharp, Bradley P. Sutton, Erick J. Paul, Nikolai Sherepa, Charles H. Hillman, Neal J. Cohen, Arthur F. Kramer, Ruchika Shaurya Prakash, Wendy Heller, Eva H. Telzer, Aron K. Barbey

**Affiliations:** 1 University of North Carolina at Chapel Hill, Chapel Hill, USA; 2 University of Illinois at Champaign-Urbana, Champaign-Urbana, USA; 3 Beckman Institute for Advanced Science and Technology, University of Illinois Urbana-Champaign, Champaign-Urbana, USA; 4 Department of Bioengineering, University of Illinois Urbana-Champaign, Champaign-Urbana, USA; 5 Northeastern University, Champaign-Urbana, USA; 6 Department of Psychology, University of Illinois Urbana-Champaign, Champaign-Urbana, USA; 7 The Ohio State University, Columbus, OH USA; 8 Carl R. Woese Institute for Genomic Biology, University of Illinois Urbana-Champaign, Champaign-Urbana, USA; 9 Neuroscience Program, University of Illinois Urbana-Champaign, Champaign-Urbana, USA; 10 Center for Brain Plasticity, University of Illinois Urbana-Champaign, Champaign-Urbana, USA

## Abstract

Although mindfulness meditation is known to provide a wealth of psychological benefits, the neural mechanisms involved in these effects remain to be well characterized. A central question is whether the observed benefits of mindfulness training derive from specific changes in the structural brain connectome that do not result from alternative forms of experimental intervention. Measures of whole-brain and node-level structural connectome changes induced by mindfulness training were compared with those induced by cognitive and physical fitness training within a large, multi-group intervention protocol (*n* = 86). Whole-brain analyses examined global graph-theoretical changes in structural network topology. A hypothesis-driven approach was taken to investigate connectivity changes within the insula, which was predicted here to mediate interoceptive awareness skills that have been shown to improve through mindfulness training. No global changes were observed in whole-brain network topology. However, node-level results confirmed *a priori* hypotheses, demonstrating significant increases in mean connection strength in right insula across all of its connections. Present findings suggest that mindfulness strengthens interoception, operationalized here as the mean insula connection strength within the overall connectome. This finding further elucidates the neural mechanisms of mindfulness meditation and motivates new perspectives about the unique benefits of mindfulness training compared to contemporary cognitive and physical fitness interventions.

## Introduction

Mindfulness meditation has increasingly been prescribed to treat psychological and mental health disorders, either accompanying traditional biological and psychological interventions or as a stand-alone treatment. The benefits of mindfulness meditation range from helping to improve the quality and duration of a cancer patient’s life^1^ to preventing the relapse of depressive episodes for those with recurrent depression^2^. Mindfulness meditation comprises a set of practices that share the core exercise of honing attention to one’s present experience without judgment^3^. To elucidate the unique benefits of mindfulness, more research is needed to specify its targets of intervention, and to compare the effects of mindfulness against those of other interventions that are thought to affect alternate (or partially overlapping) psychological functions and associated neural mechanisms.

Mindfulness meditation is known to improve a range of cognitive and emotional processes. Cognitive skills improved through mindfulness include sustained attention and working memory capacity^4^. Under effectively neutral conditions, such improvements in attention and working memory overlap with gains resulting from other types of cognitive training^5^. In clinical and non-clinical adolescent and adult populations, mindfulness training has been shown to reduce levels of depression, anxiety, and trait negative affect, an associated personality dimension related to neuroticism^6,7^. Unlike other forms of emotion regulation such as cognitive reappraisal^8^, which leverage higher cortical regions to consciously alter evaluations of one’s emotional state, mindfulness may improve emotional responding via a strengthening of attention to visceral, sensory representations of emotion^9^. Indeed, allocating more awareness to interoceptive sensations involved in emotional states through mindfulness practices and less to thoughts evaluating the emotion has been shown to reduce the intensity and improve regulation of negative emotional states^10^. Moreover, several disorders marked by a lack of emotion awareness and regulation, such as alexithymia and autism, show deficits in mechanisms of interoceptive awareness primarily in insula cortex^11^.

Such psychological models of change have recently been investigated using the tools of neuroscience. In a recent, comprehensive review^12^ it was reported that mindfulness training produces several functional and structural neural changes associated with various psychological functions. A whole-brain examination of white matter changes over short-term mindfulness training found increases in fractional anisotropy (FA; a measure of structural integrity of axonal fiber bundles related to myelogenesis) in tracts broadly involved in emotional processing and attention^13^. Converging evidence demonstrates that mindfulness training increases emotion regulation and FA within the uncinate fasciculus, a fiber bundle connecting regions within temporal cortex (lateral temporal gyri, insula) and subcortical structures involved in emotion (amygdala) to cognitive control areas within orbitofrontal and medial prefrontal cortices^14^.

The endeavor to understand how mindfulness works will likely be advanced by using recently developed tools and theory within the nascent field of brain connectomics. The connectomic framework conceives of the brain’s functional and structural architecture as a complex, dynamic network. This network view of brain function partly arose from the lack of support for highly selective, modular regions instantiating specialized functions^15^. That is, large meta-analyses consisting mostly of univariate fMRI analyses disconfirm that, for example, the amygdala is exclusively selective for fear processing. Indeed, more fruitful mechanistic knowledge^16^ of how neural systems function may emerge from delineating how different regions communicate functionally across a range of environments, and by identifying the underlying structural connections that constrain such functional dynamics^17,18^.

Although there have been several studies using network-related methods (i.e., functional and structural connectivity) to study the effect of mindfulness training, few have explicitly leveraged graph-theoretical methods (e.g., global efficiency, mean node strength) to probe connectome changes associated with mindfulness training. One recent graph-theoretical study has found increased functional degree centrality changes in insula^19^. The study’s intervention, however, included a combination of fitness training and nutrition guidance, examined a relatively small sample (*n* = 31), and compared mindfulness training to a waitlist control, together limiting the ability to infer the unique changes induced by mindfulness.

In the present study, patterns of structural connectome changes induced by mindfulness training were compared to a control group and demonstrated to be additive to other cognitive and fitness training across both groups (Table 1).

**Table 1:** Intervention schedule.

	Pre-Assessment (3–4 sessions) Weeks 1–2	Intervention (48 sessions)						Post-Assessment (3–4 sessions) Weeks 19–20
		Weeks 3–5	Week 6	Weeks 7–12		Weeks 13–18		
Cognitive Training + Fitness	Demographics Cognitive testing Accelerometer Diet record Fitness assessment Neuroimaging	Fitness	Fitness	Fitness+	Cognitive	Fitness+	Cognitive	Demographics Cognitive testing Accelerometer Diet record Fitness assessment Neuroimaging
Cognitive Training + Fitness + Mindfulness		Mindfulness	Mindfulness+	Fitness	Fitness	Fitness+	Cognitive	

We hypothesized that mindfulness training would produce changes in neural regions associated with interoception. Using an active control that affects similar processes to mindfulness (e.g., cognitive control) allowed us to infer that changes in insula networks required mindfulness training, which unlike the active control, included interoceptive training (e.g., focusing on inner bodily states). Indeed, mounting evidence has demontrated that mindfulness alters interoceptive capacity, although mechanisms that may account for this effect remain elusive^20–24^. This evidence suggests that although mindfulness may not increase the *accuracy* of perception to interoceptive signals, attention, identification, and proper utilization of interoceptive signals for self-regulatory purposes have been shown to increase through mindfulness^21^.

Interoception is the perception of the internal physiological state of the body, including sensations such as pain, thirst, hunger, and sexual arousal^25^. Distinguished neuroanatomically from proprioception and exteroception pathways^26^, interoception features prominently in theories of emotion generation, awareness, and regulation. Many have argued and demonstrated that (1) changes in physiology can initiate the cascade of emotional experience and (2) the capacity to perceive shifts in the physiological state of one’s body relates to the capacity to sense and regulate emotional episodes^27^.

To optimize our sensitivity to selectively detect connectivity changes from mindfulness training, we focused on connections with regions demonstrated to be involved in interoception. To identify these regions, we consulted the existing literature on mechanisms involved in interoception as well as reverse-inference maps from NeuroSynth (which describe the likelihood of a psychological term appearing in a paper given the location of brain activity; 15). Two regions, insula cortex and anterior cingulate cortex, have been consistently associated with interoception in both human functional neuroimaging experiments as well as post-mortem histological studies of humans and non-human animals^25^. Given that interoception is not the only function such neural regions are involved in, we used NeuroSynth to determine which of these regions were least associated with cognitive control and working memory, two processes known to be improved by the active control in the present study. Reverse-inference maps revealed that insula cortex had a much stronger association (~0.8 posterior probability) with interoceptive awareness, and weaker association with cognitive control (~0.3), whereas anterior cingulate cortex showed the opposite pattern. Such functional neuroimaging meta-analytic data comports with extant literature, including structural neuroanatomical findings, which predominately emphasizes insula cortex in interoception and interoceptive awareness^26–28^.

**Figure 1:**
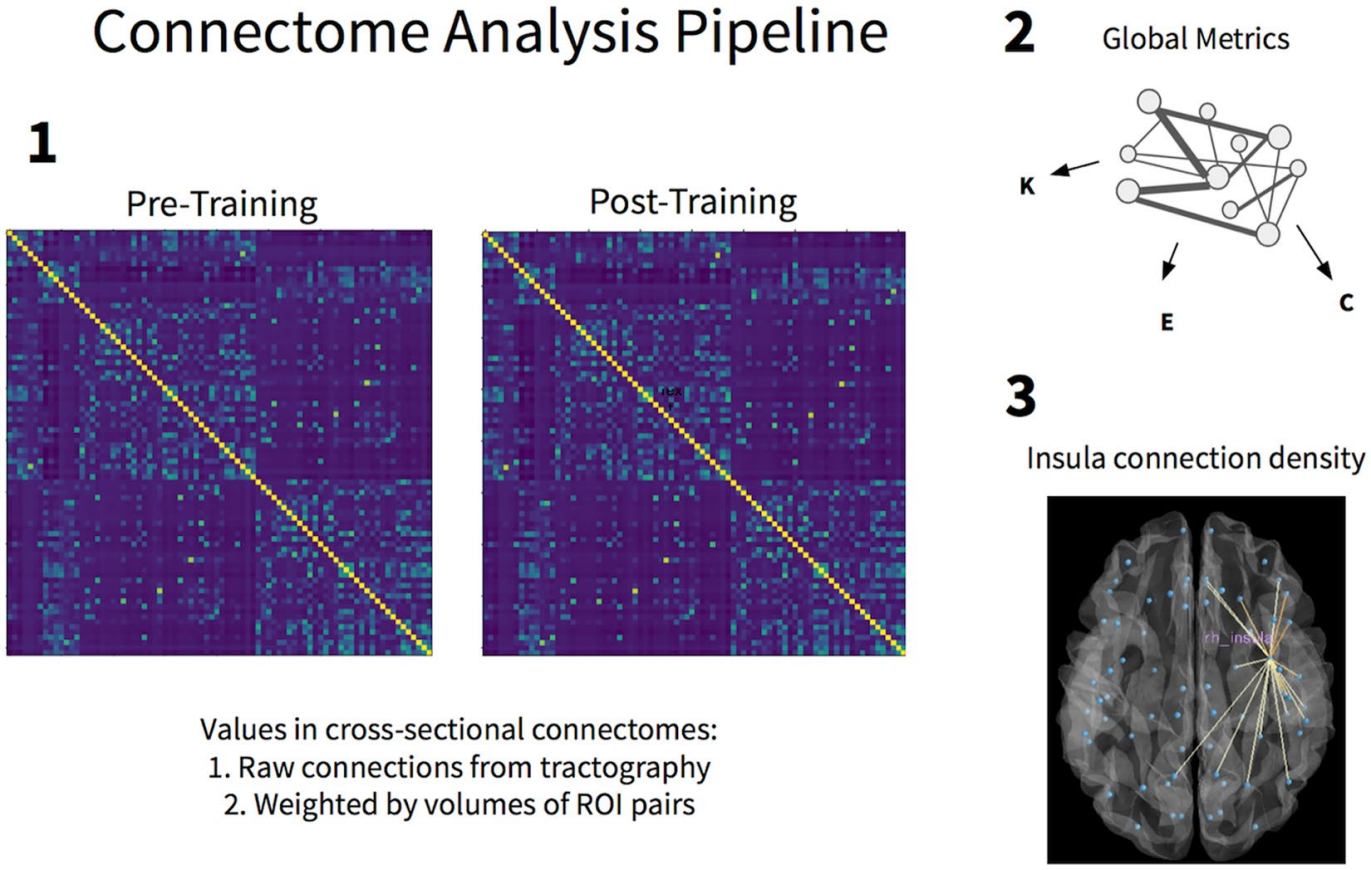
Overview of the connectome analysis for mindfulness training. (1) Connectomes are obtained from pre- and post-training data. Each entry in the connectome matrix contains connection strength for a given connection (e.g., insula to OFC), which is computed by dividing the raw number of tracks from tractography by the average volume of each pair of ROIs. (2) Three global graph-theoretical metrics were computed on the overall connectome: mean strength (**B**), global efficiency (**E**), and mean clustering coefficient (**C**). (3) Connection density (i.e. connection strength) was summed across left insula and right insula, yielding two summary metrics of left and right insula mean connection density.

Two connectomic analyses (Fig. 1) were employed to investigate structural neuroplasticity across experimental groups: (1) graph-theoretical changes in global network topography and (2) connection-of-interest (COI) changes in insula circuits. Objective (1) was implemented by a hypothesis-free exploration of global changes that may be induced by mindfulness training. For objective (2) we hypothesized that insula strength, defined by the connection density of right or left insula across all connections, would be greater for the mindfulness group compared to the active control group. Importantly, using connection strength changes to measure neuroplasticity has recently been validated empirically as a marker of clinical changes^29^.

## Methods

### Participants

All participants in the present analysis were selected from a larger multimodal intervention study (full information in Supplemental Material). The present analysis included only participants assigned to the cognitive training + fitness + mindfulness training and cognitive training + fitness (to serve as a control/comparison group).

A total of 113 and 114 participants were initially recruited in each group, respectively. Approximately 75% of each group received an MRI scanning session and of those, 45–50% remained enrolled through study completion. Eighty-six participants were included in present analyses: 43 from the cognitive training + fitness comparison group, and 43 from the cognitive training + fitness + mindfulness experimental group.

All enrolled participants were screened for the following eligibility criteria: aged 18–44; BMI <35; right-handed; normal or corrected-to-normal vision without color blindness; no previous neurological injuries, disorders, or surgeries; no medications affecting central nervous function (including >10 cigarettes per day); not pregnant; no head injury or loss of consciousness in the past 2 years; and proficient in English. All participants signed informed consents. The University of Illinois at Urbana-Champaign Institutional Review Board approved all procedures used (IRB14212), and all methods were performed in accordance with the relevant guidelines and regulations. All participants signed informed written consents before participation in the study.

Attrition, due to withdrawal from the study or incomplete data for MRI analyses, was highly similar across the two groups used in the present analysis (active control: 49.4%, mindfulness group: 48.8%; See Table S1 in Supplemental Material). High attrition was likely due to the following factors: (1) the length of the intervention, (2) conflict with the academic calendar (the intervention sometimes spanned holidays, campus breaks, final exams, etc.), (3) the complex work schedule of college undergraduates, (4) the highly demanding experimental tasks, and (5) the requirement to gain competence in multiple intervention modalities (e.g., fitness, cognitive, and/or mindfulness training).

### Procedure

Study participation lasted over a 20-week period. Weeks 1–2 included collection of demographics, pre-assessment cognitive testing sessions, an 8-day accelerometer recording, a 7-day diet record, a fitness assessment, and a neuroimaging (MRI) session. Weeks 3–18 comprised intervention training, during which participants completed 210 minutes of training per week, divided into three 70-minute sessions. The cognitive training + fitness + mindfulness group completed a total of 10 cognitive training sessions, 28 fitness sessions, and 10 mindfulness sessions. The cognitive training + fitness group completed a total of 20 cognitive training sessions and 28 fitness sessions. Weeks 19–20 included post-assessment measurements matching those administered in the pre-assessment. This schedule is summarized in Table 1.

### Interventions

Both groups took part in the physical fitness intervention, and cognitive training. Fitness training included a combination of low and high intensity cardiovascular and weight training (summarized fully in Supplemental Material). Cognitive training comprised Mind Frontiers, a suite of cognitive training games developed in collaboration with Aptima, Inc. The Wild West-themed game primarily consists of seven mini-games that were derived from cognitive exercises that have been previously demonstrated to target cognitive abilities and/or to improve cognitive performance^30–35^. Those abilities include: processing speed^36,37^, attention^36,37^, working memory^38^, relational memory^39^, cognitive flexibility^40^, and problem-solving^41^. Previous research has suggested that integrative training across these skills will promote greater enhancements in fluid intelligence than training on any single paradigm^32,42^. Mind Frontiers also includes a meta-game to increase player motivation and engagement in the mini-games.

Participants in the mindfulness group practiced for a total of 11.67 hours, comprising ten 70-minute session. Active researchers and practitioners of mindfulness meditation were consulted to aid in constructing the intervention. The intervention emphasized core tenants of mindfulness (e.g.,^43^), such as learning to recognize internal and external sensations as well as thoughts arising in consciousness with nonjudgment. In each mindfulness meditation training session, participants completed breathing exercises, a period of guided meditation, and two periods of silent meditation. During the meditation period, a soft chime rang every five minutes to reorient participants to their practice. Between the two silent meditation periods, some gentle movement and stretching was incorporated to reduce the risk of discomfort caused from remaining seated for a sustained period of time. A typical mindfulness meditation training session is described in Table 2.

**Table 2:** Summary of a 70-minute mindfulness meditation training session design.

Phase	Instructions	Duration
Questionnaires	PANAS, MAAS, FFMQ, Flow State Scale, M-CER, and/or MiniMASQ	5 min
Breathing Exercises	Promotes mindfulness of breathing, focuses attention, reduces mind-wandering and distraction	5 min
Guided Meditation	Promotes observation and nonjudgment of thoughts, teaches mindfulness meditation techniques to apply in silent meditation	5 min
Gentle Movement/Stretching	Promotes mindfulness of body movement, prepares body for meditation	15 min
Silent Meditation	Independent mindfulness-based meditation	30 min
Individual Q & A	Between participants and instructor	10 min

The only exception to this protocol was in the first introductory training session, which contained more verbal instruction, a Q & A period, and only one period of silent meditation. The total duration and structure of the present mindfulness training comports with previous brief mindfulness training programs^13^. To validate that the present mindfulness intervention was successful, the Mindful Awareness and Attention Scale questionnaire was administered before and after training. As expected, significant gains occurred between the first to last session of training (t(55) = 4.45, p < 0.001^44^;).

Importantly, to be included in the present analyses, participants had to complete at least 90% of the sessions, i.e. at least 9 out of 10 mindfulness sessions. Thus, all participants completed at least 10.5 hours of mindfulness training, which is comparable to previous forms of mindfulness training that have been shown to induce both functional and structural brain changes^12^.

### Self-Reported Interoception

We used the Five Facet Mindfulness Questionnaire – Observe subscale to measure self-reported interoception^45^. The purpose of using this measure was to determine if insula changes related to increases in subjective awareness of interoception. Questions on this subscale emphasize interoceptive awareness, such as, “When I’m walking, I deliberately notice the sensations of my body moving” or ones focused on emotions, such as, “I pay attention to how my emotions affect my thoughts and behavior.”

### Imaging acquisition

Diffusion weighted imaging (DWI) data was collected using a Siemens 3 T Trio MRI scanner and a 32-channel head coil. The acquisitions consisted of 30-direction DWI data with a b-value of 1000 s/mm^2^ and 2 b = 0 s/mm^2^ images acquired at the beginning of the run. The imaging consisted of 72-slices, 2 mm thick acquired with 1.9 mm × 1.9 mm in-plane resolution. A single-shot, spin-echo EPI acquisition was used with TE of 100 ms, TR of 5 s, an SMS multiband factor of 2 (https://www.cmrr.umn.edu/multiband/)^46–49^ and a GRAPPA factor of 2 for parallel imaging^50^. In addition to the DWI scan, a structural T1-weighted magnetization-prepared rapid acquisition of gradient echo (MPRAGE) acquisition was acquired with 0.9 mm isotropic resolution, TE of 2.32 ms, TR of 1.9 s, and a magnetization preparation pulse with an inversion time, TI, of 900 ms.

### Preprocessing

Prior to connectome reconstruction, diffusion weighted data was preprocessed by converting DICOM files to NIFTI format, followed by eddy current correction using an affine registration to the b = 0 image (i.e. without gradients). Finally, in preparation for probabilistic tractography, FSL’s bedpostx^51^ was run, which estimates a probability distribution of primary fiber orientations at each voxel using Markov chain Monte Carlo sampling. All computation was carried out on AWS NITRC-CE instances (http://www.nitrc.org).

### Cortical Parcellation

Freesurfer’s recon-all^52^ was run on each subject’s high-resolution T1-weighted structural image. This generated a cortical parcellation from which regions were defined for subsequent probabilistic tractography. The present analysis used the 68 cortical regions defined by Freesurfer that cover the entire cortex, and 14 from subcortical regions, which comprise an 82 region connectome. To prepare these regions for tractography, each region was registered to diffusion-weighted space, first using Freesurfer’s bbregister tool (using FSL’s FLIRT initialization) to compute the transformation matrix from diffusion-weighted space to T1 space. This was followed by Freesurfer’s mri_vol2vol to bring the Freesurfer parcellations into diffusion-weighted space using the inverse of the previously computed transformation matrix. Bbregister has been shown to improve registration beyond more traditional methods, in which the cost function examines gradient directions and magnitudes across tissue boundaries^53^.

### Probabilistic Tractography

FSL’s probtrackx2^51^ was used to carry out probabilistic tractography. Five thousand “seed” streamlines were generated from each voxel within each of the 82 regions, and targets were defined as any voxel within the 81 additional regions. Using protrackx2 in network mode, the output included a matrix, fdt_network_matrix, which contained the number of streamlines from each seed volume (e.g., all voxels in insula cortex) that reached all other 81 target regions. We also included all ventricles in the avoid option, which exclude tracks that go through the ventricles and are thus physically implausible. All other options for probtrackx2 were set to default inputs.

### Connectome Reconstruction

Each entry in the connectome was normalized by the average volume of each ROI comprising the pathway. This method of weighting is a modified version of the connectome density function^54^, in which the normalization for track length was not included. We note that normalization by track length is only appropriate for tractography methods that seed white matter voxels, whereas our approach seeds from ROI volumes in gray matter. Moreover, because the present connectomes are weighted as opposed to binarized, no thresholding of tracks was carried out^54,55^. Connectomes were then symmetrized, by averaging identical entries that were initiated from opposite ends (e.g., amygdala to insula and insula to amygdala). This was done for two reasons: (1) the graph-theoretic algorithms require symmetric matrices and (2) one cannot determine whether axonal bundles comprise efferent or afferent pathways^56^ reconstructed from diffusion-weighted data.

### Graph-theoretical Analyses

Connectomes comprise two basic components: nodes and edges. Nodes, in this study, are gray-matter regions defined from the parcellation, and the edges are the weighted strength of each pathway between nodes. To carry out subsequent graph-theoretical analyses, a Python implementation, bctpy^57^ of the Brain Connectivity Toolbox^58^ was applied.

Three graph-theoretical metrics were computed on both pre-training and post-training connectomes. These metrics have been shown to be reliable across time using the same pipeline we followed to reconstruct connectomes^59^. Measures included mean strength (K), global efficiency (E), and mean clustering coefficient (C). K is the average of connection strengths across all nodes, and thus represents global structural connectivity. To compute graph-theoretical measures of *topological* distance, we transformed the original connectome into a connection-length matrix. Connection-length matrices are computed as the element-wise inverse of connection strengths. In graph theory, stronger connections are considered to be more proximal, regardless of the underlying physical distance. E is computed as the average inverse topological distance between nodes, and is a measure of integration. C indicates the average density of clustering for a given node, and is a measure of modularity or network segregation^59^. Thus, selected graph-theoretical measures comprised indicators of overall network integration (K,E) and segregation/modularity (C). Finally, for the node-level analysis, the mean connection strength was computed for both left and right insula (e.g., the sum of all 81 entries in the connectome involving right insula). This measure defines to what degree insula, which has been shown to be a hub region^60^, can communicate efficiently with all other regions within the overall network.

### Statistical Analyses

To measure whether mindfulness impacted each *a priori* defined outcome measure (3 global graph-theoretical metrics and 2 insula-specific metrics), we conducted 5 ANCOVAs in which the experimental group was the fixed factor predicting post-training outcomes controlling for pre-training outcomes.

## Results

### Graph-theoretical Global Measures

No significant differences were found between the mindfulness training group and the comparison group across the three measures of whole-brain network topology. Out of the 3 ANCOVAs run on each graph-theoretical metric, none approached significance (e.g., the largest non-significant result was for mean strength across all nodes: f = 0.2, p = 0.653).

### Connection of Interest Analyses

**Figure 2:**
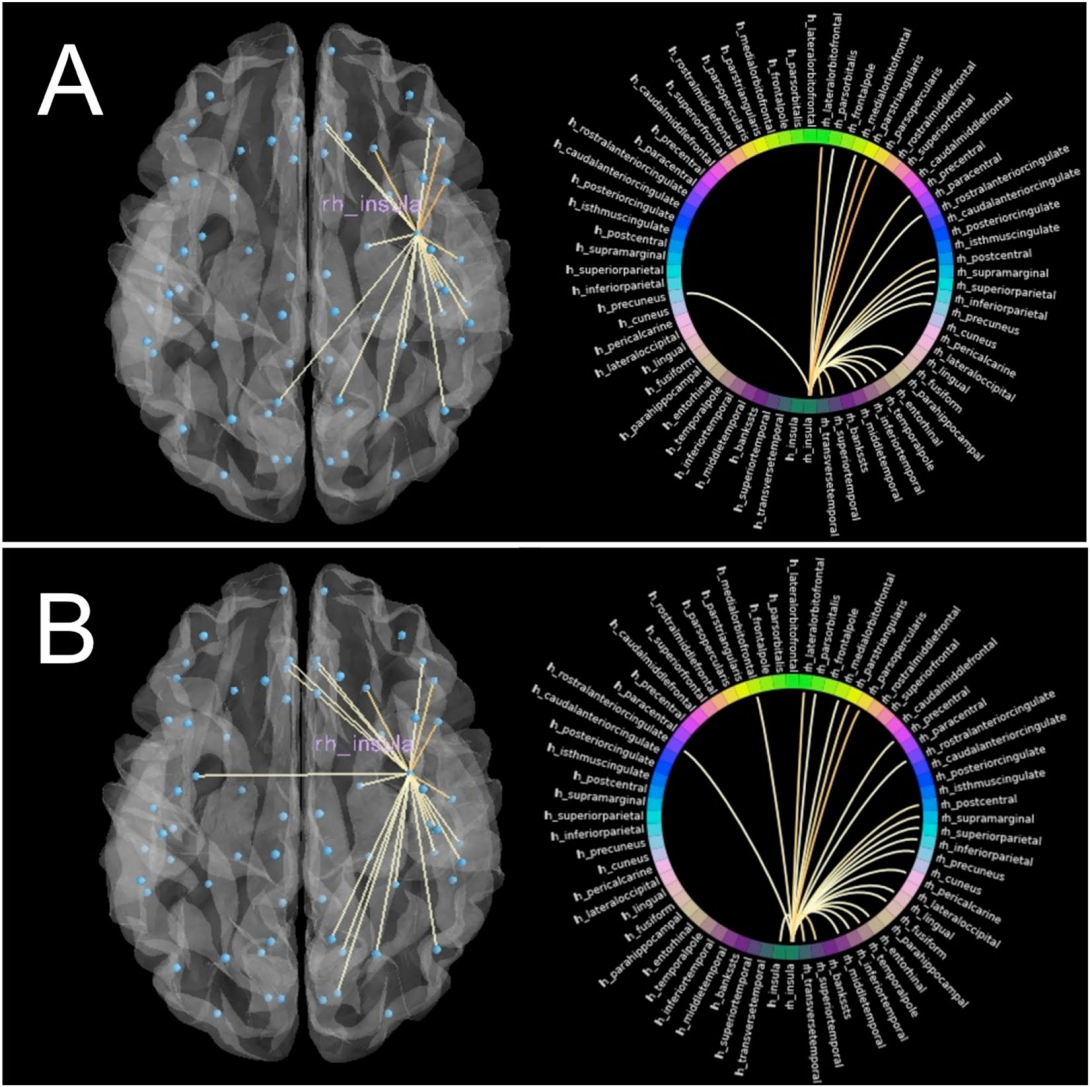
In both panels, the left brain-image is an anatomical representation of tractography pathways between right insula and highly connected regions, and the right diagram is a graphical representation of those same right insula connections. Connections displayed (only corticocortical, here) comprised the top 80% connection strengths across all insula pathways. (**A**) Displays pre-training connections in right insula, which showed the greatest structural reorganization across mindfulness training. (**B**) Represents the same two images as in (**A**) except at post-training.

An ANCOVA was run on post-training mean strength of insula controlling for pre-training differences in mean insula. An ANCOVA was carried out as opposed to a repeated measures ANOVA because there were significant differences at Time 1 in total insula strength across groups despite randomization (t(81) = 1.98, p = 0.05), which is controlled for in the present ANCOVA design. This procedure has been suggested as the preferred method for randomized controlled trials (as is the present study), as it has been empirically demonstrated that for established true effects, ANCOVAs controlling for baseline differences are more powerful to detect these effects than repeated measures ANOVA^61^. Three subjects were excluded from the analyses because the absolute value of their insula connection strength was greater than 2.5 standard deviations from the mean. The mindfulness group demonstrated an increase in mean strength within right insula at post-training (F1,80 = 4.729, p = 0.03), which approached a medium effect (η^2^_partial_ = 0.056). For left insula, results were non-significant. To depict the change in right insula connections, connectomes from each time-point from a single, representative participant in the mindfulness group is displayed in Fig. 2.

Follow-up one sample t-tests on difference scores (post-training minus pre-training insula connections compared against a change of 0) reflect that only the mindfulness training group (t = 2.45, p = 0.019) and not the cognitive training group (t = 0.97, p = 0.334) had sufficient gains in insula under the presumption of the null hypothesis that there were no gains. Gains in insula connectivity were unrelated to gains in self-reported interoceptive awareness for the mindfulness group (*r* = −0.13, p = 0.42).

## Discussion

The present study is the first to examine the unique structural connectome changes induced by mindfulness training. Results partially supported *a priori* hypotheses in that only the mindfulness group had node-level changes in the structural reorganization of right insula. Additionally, in whole-brain comparisons across experimental groups, mindfulness was not shown to shift global network-wide properties related to network integration, segregation, and the influence of hub nodes. This analysis demonstrated specific effects of mindfulness training on the insula network.

Increased connection strength found in right insula comports with studies showing lateralization in insula, in which right insula is more strongly associated with emotion awareness^28^. Moreover, since underlying structural dynamics (controlled for inter-regional distance) correlate highly with resting-state functional dynamics^62^, it is likely that the observed structural changes in insula result in functional changes. Thus, present findings may provide a mediating pathway by which mindfulness practice induces graph-theoretical functional connectivity reorganization in insula networks^19,20^. Future work should endeavor to further link the functional and structural changes in insula that result from mindfulness training.

In addition to its association with interoception, insula is associated with emotion awareness. Conceptually, interoception and emotional processing are closely related phenomena, wherein some consider interoception to be necessary for all emotional episodes^63^. To a first approximation, the conceptual link between interoception and emotion is intuitive, in which interoception-mediated states such as hunger and pain possess affective dimensions of valence and arousal that differ from baseline core affect^64^. Such work has been advanced recently using predictive coding models of brain function, which suggest that the mismatch (called prediction error) between sensory-driven interoceptive signals (and sometimes exteroceptive signals as well) with top-down generative models of what such signals should be may be part of the explanation of emotional phenomena^65^. Some argue that the most evolutionary-developed region of insula that is unique to humans – the anterior insula cortex – is vital to integrating information from interoceptive and exteroceptive signals via iterative updating of generative models^27^. Within the dynamic, unfolding process of matching interoceptive predictions (e.g., “my body is in a safe place”) and exteroceptive predictions (e.g., “the environment is safe”) with bottom-up sensory information (e.g., what the sensorium detects when “a bear is impinging upon me”), it is the resultant prediction errors that may be necessary to generate emotional episodes (e.g., fear) and in some cases spur actions to minimize prediction error by changing the environment (e.g., fleeing/fighting). As such, anterior insula in particular has a quite broad functional architecture that may be involved in several possible pathways to emotion generation (e.g., social conflict or physical pain).

The present findings help specify the targets of intervention of mindfulness meditation, which can inform clinical decisions related to treatment. Pathologies marked by deficits in interoception and emotion awareness may be most benefitted by mindfulness interventions. Deficits in interoception, as measured physiologically through heartbeat perception, are associated with major depression^66^. From a transdiagnostic perspective, self-reported interoceptive awareness has also been shown to improve emotion regulation via improved cognitive reappraisal of aversive stimuli^67^. Future work should assess whether gains in insula reorganization due to mindfulness may be indicative of improved emotional awareness, given the association between anterior insula and emotional awareness^25,28^ as well as demonstrations that mindfulness improves self-reported ability to better differentiate between emotional states^68^. This will be contingent on more fine-grained parcellations of insula cortex, in which anterior insula should be defined as a separate node due to its stronger association with emotion awareness^25^. Like interoceptive awareness, emotional awareness has been shown to predict deficits in emotion regulation strategies, and as such, may help a range of psychopathology that’s marked by poor emotion regulation.

In closing, we note several limitations of the present study. First, the underlying anatomical meaning of “connection strengths” in weighted connectomes has not been well established in the field and depends on many unrelated sources of variance, including even the diffusion-weighted imaging protocol^56^. However, the present *longitudinal* design offers a unique opportunity to use this measure more appropriately. Because many sources of variance are held constant within individuals and within a given pathway between time-points, it is likely that differences in connection strength relate to meaningful microstructural differences, such as increases in myelination or fiber count. Indeed, it has been shown that various short-term forms of cognitive training (between 2 and 10 hours) have been shown to induce changes in white matter correlates using DTI^13,69^. Moreover, it has been demonstrated that short-term neuronal stimulation, characteristic of changes induced by short-term cognitive training, promotes long-term myelogenesis in mice^70^.

Second, because the mindfulness manipulation also included fitness and cognitive training, and because the cognitive training in the mindfulness group differed in duration relative to the comparison group, results may reflect interactions between such training and mindfulness, or may be due to differences in length of cognitive training. The mindfulness group had 10 mindfulness training sessions and 10 cognitive training sessions whereas the active control group had 20 cognitive training sessions. To establish the unique contribution of each intervention modality, future studies should systematically compare groups that each comprise only one type of training. Third, the present results do not compare the effects of cognitive processes known to be affected by mindfulness, such as cognitive control, and as such do not resolve whether or not mindfulness improves mechanisms of cognitive control above other forms of cognitive and fitness training. Indeed, increased insula connectivity may be related to the improvement of other cognitive processes, such as cognitive control or exteroception.

Lastly, we did not have more direct measures of interoception, such as heartbeat perception, to associate with gains in insula connectivity. Future studies should endeavor to theoretically advance and experimentally implement behavioral measures of interoception^21^. Although we did not find that insula connectivity was related to a measure of self-reported interoceptive awareness, interoception is difficult to assess (especially gains below conscious awareness) with self-report and behavioral measures, and consensus has not been reached in the field about how it should be measured^14,21^. Moreover, it should be noted that a more recent questionnaire designed to assess interoceptive awareness found that attention to bodily signals does not increase over mindfulness (which is what the measure we included focused on), but what does change is how body sensations are used to regulate behavior^71^. Thus, we contend that measuring insula connectivity after mindfulness training that engages mechanisms for interoception (e.g., body awareness) is one such way to measure and characterize gains in interoception.

The present findings establish how mindfulness induces structural connectivity changes beyond intensive physical fitness and cognitive training. The gain in insula connection strength only for the mindfulness group is suggestive of increase in the capacity to perceive internal experience. Moreover, previous work suggests that those who benefit most from mindfulness have either high levels of stress^72^ or psychopathology^1^. Thus, the present effects may be even more pronounced in clinical or high-stress samples. Future work should leverage multiple neuroimaging modalities with increased anatomical and temporal sensitivity to further explicate the mechanisms of interoception which appear to change through mindfulness training.

## Electronic supplementary material

Supplemental material
