# Supplementary material for: Mindfulness training induces structural connectome changes in insula networks
Source: Sci Rep. 2018 May 21;8:7929. doi: 10.1038/s41598-018-26268-w (PMC5962606; doi:10.1038/s41598-018-26268-w)
Supplement: Supplementary file 1 — Supplemental material [file 41598_2018_26268_MOESM1_ESM.docx]

**Mindfulness training induces structural connectome changes in insula networks supplemental information**

Paul B. Sharp­^1^, Bradley P. Sutton^2,3,4^, Erick J. Paul^2^, Nikolai Sherepa^2,3^, Charles H. Hillman^5^, Neal J. Cohen^2,3,6^, Arthur F. Kramer^2,5^, Ruchika Shaurya Prakash^7^, Wendy Heller^2,3,6^, Eva H. Telzer^1^, & Aron K. Barbey^2,3,4,6,8,9^

^1^University of North Carolina at Chapel Hill, ^2^University of Illinois at Champaign-Urbana, ^3^Beckman Institute for Advanced Science and Technology, University of Illinois Urbana-Champaign, ^4^ Department of Bioengineering, University of Illinois Urbana-Champaign, ^5^Northeastern University, ^6^Department of Psychology, University of Illinois Urbana-Champaign, ^7^The Ohio State University, ^8^Carl R. Woese Institute for Genomic Biology, University of Illinois Urbana-Champaign, ^9^Neuroscience Program, University of Illinois Urbana-Champaign

Keywords: *Connectomics, mindfulness, interoceptive awareness, cognitive training, neuroplasticity*

Address for Correspondence:

Paul Sharp; E-mail: [psharp89@live.unc.edu](mailto:psharp89@live.unc.edu)

Aron K. Barbey; E-mail: [barbey@illinois.edu](mailto:barbey@illinois.edu)

Decision Neuroscience Laboratory

Beckman Institute for Advanced Science and Technology

University of Illinois at Urbana-Champaign

**Detailed Participant information**

A total of 453 participants were recruited and randomly assigned either to one of three experimental groups or an active control group. The experimental groups consisted of: fitness training only; cognitive training and fitness (cognitive training + fitness); and cognitive training, fitness, and mindfulness meditation (cognitive training + fitness + mindfulness). The active control group performed active visual search and change detection tasks [1,2].

All enrolled participants were screened for the following eligibility criteria:aged 18-44; BMI < 35; right-handed; normal or corrected-to-normal vision without color blindness; no previous neurological injuries, disorders, or surgeries; no medications affecting central nervous function (including >10 cigarettes per day); not pregnant; no head injury or loss of consciousness in the past 2 years; and proficient in English. The University of Illinois Urbana-Champaign Institutional Review Board approved all aspects of this study.

**Detailed Fitness Training Information**

Both groups took part in the physical fitness intervention, which was conducted in a group setting of up to 20 participants per class. Participants in each class were divided into groups of 5 or fewer participants per trainer. During the fitness sessions, participants’ heart rate was monitored using a Polar heart rate monitor (Polar E600, Polar Electro, Finland). Each group completed a total of 28 fitness training sessions (see Table 1).

Each fitness training session was composed of a warm-up (10 minutes), a light walk/run (10 minutes), high intensity cardio and resistance (30 minutes), whole-body drills (10 minutes) and a cool-down (10 minutes). The warm-up was the same at every session and included dynamic stretches and light activity to prepare the body for exercise. The Walk/Run portion varied in time and distance among the sessions. The high intensity cardiovascular resistance training (HICRT) was split into two segments of three sets of three-to-four exercises. Participants completed one to two minutes of jump rope and a four-minute power series between the two segments. The HICRT exercises varied among sessions and included exercises involving bodyweight, resistance bands, kettlebells, body bars, and suspension training. The drills portion also varied among sessions and included whole-body training with equipment such as battle ropes, sand bags, ladders, medicine balls, and parachutes. Every fitness session finished with yoga-inspired stretching and a cool-down routine.

To assess the impact of fitness training on each participant’s physical fitness, the Army Physical Readiness Test (APRT) was completed at the first and last session of the 28-session fitness intervention. The test includes five exercises: (1) 60-yard shuttle run, (2) standing long jump, (3) one-minute of rowers, (4) one-minute of push-ups, and (5) a 1.5 mile run.

**Table S1. Participant recruitment and attrition**

**
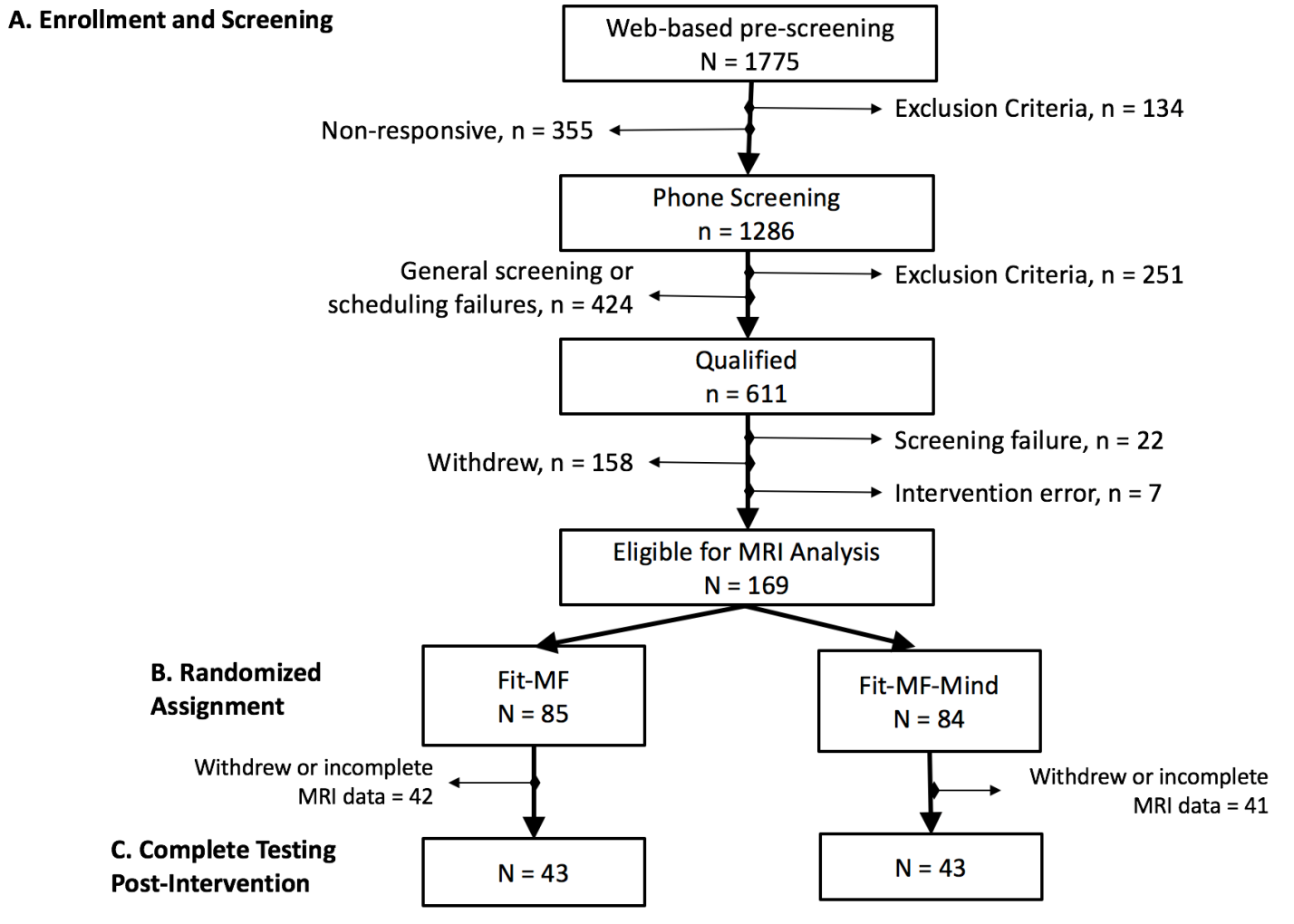
**

**References**

1. Gaspar, J. G., Neider, M. B., Simons, D. J., McCarley, J. S., & Kramer, A. F. (2013). Change Detection: Training and Transfer. PLoS ONE, 8(6), e67781. <http://doi.org/10.1371/journal.pone.0067781>
2. Harrison, T. L., Shipstead, Z., Hicks, K. L., Hambrick, D. Z., Redick, T. S., & Engle, R. W. (2013). Working Memory Training May Increase Working Memory Capacity but Not Fluid Intelligence. Psychological Science, 0956797613492984. <http://doi.org/10.1177/0956797613492984>
